# Mass production of low-boiling point solvent- and water-soluble graphene by simple salt-assisted ball milling†

**DOI:** 10.1039/c9na00463g

**Published:** 2019-11-21

**Authors:** Yoshihiko Arao, Riichi Kuwahara, Kaoru Ohno, Jonathon Tanks, Kojiro Aida, Masatoshi Kubouchi, Shin-ichi Takeda

**Affiliations:** Tokyo Institute of Technology, School of Materials and Chemical Technology 2-12-1 O-okayama, Meguro-ku Tokyo Japan yoshihiko.arao@gmail.com; Dassault Systèmes ThinkPark Tower 2-1-1 Osaki, Shinagawa-ku Tokyo Japan; Department of Physics, Yokohama National University 79-5 Tokiwadai, Hodogaya-ku Yokohama Japan; National Institute for Materials Science Sengen 1-2-1, Tsukuba Ibaraki Japan; Structures and Advanced Composite Research Unit, Japan Aerospace Exploration Agency (JAXA) 6-13-1 Osawa, Mitaka-shi Tokyo Japan

## Abstract

Developing a mass production method for graphene is essential for practical usage of this remarkable material. Direct exfoliation of graphite in a liquid is a promising approach for production of high quality graphene. However, this technique has three huge obstacles to be solved; limitation of solvent, low yield and low quality (*i.e.*, multilayer graphene with a small size). Here, we found that soluble graphite produced by mechanochemical reaction with salts overcomes the above three drawbacks. Soluble graphite was exfoliated into monolayer graphene with more than 10% yield in five minutes of sonication. The modified graphite was easily exfoliated in a low-boiling point solvent such as acetone, alcohol and water without the aid of a surfactant. Molecular simulation revealed that the salt is adsorbed to the active carbon at the graphite edge. In the case of weak acid salts, the original bonding nature between the alkali ion and the base molecule is retained after the reaction. Thus, alkali metals are easily dissociated in a polar solvent, leading to negative charge of graphene, enabling the exfoliation of graphite in low boiling point solvents. The approach proposed here opens up a new door to practical usage of the attractive 2D material.

## Abstract 2

The salts adsorbed at the graphite edge facilitate the exfoliation of graphite in liquid.

## Graphical abstract

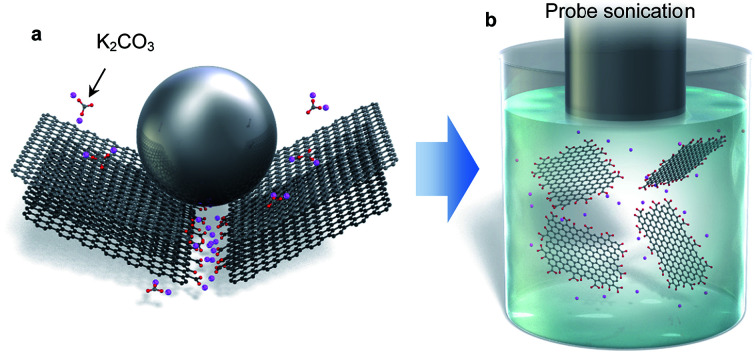

## Introduction

Graphene has received enormous attention in the field of microelectronics and composite materials. A wide range of applications such as high-sensitivity sensors, thin film transistors, transparent conductive films, and anti-corrosion coatings have been proposed up to now.^1–6^ Commercialization of graphene for these attractive applications highly depends on the progress of graphene production technology. Bottom-up approaches like chemical vapor deposition can fabricate large-area high-quality graphene, but the productivity is usually at the milligram-scale and it is not likely to become a mainstream mass production technique.^7^ Thus, a top-down approach—*i.e.*, exfoliation of graphite—is the only feasible method to produce graphene at the ton scale.

Graphene was first produced by mechanical cleavage using scotch tape to literally peel off layers from natural graphite.^8^ Of course, this method requires considerable labor and is not appropriate for mass production. It is well known that several atomic or molecular species can be chemically inserted between graphene layers of the host graphite, a process known as intercalation. Graphite intercalation compounds (GICs) can be produced by means of anodic or chemical oxidation; in general, 0.11 wt. equiv. of potassium permanganate (KMnO_4_) is added into concentrated sulfuric acid (98%) to covert 1 wt. equiv. of graphite to stage 1 GICs.^9^ Expanded graphite is produced by intercalation of a strong acid following gasification of the intercalant.^10–12^ After that, the expanded graphite is exfoliated to produce multi-layer graphene (>10 layers). This method is the simplest and its feasibility for mass production has already been established. However, there are no repulsive forces between the platelets in dry powder form (*i.e.*, no solvent), only attractive van der Waals forces. This results in agglomeration of the multi-layer graphene platelets that are difficult to disperse due to their large face-to-face contact area. When 6 wt. equiv. of KMnO_4_ is added during the intercalation process, the GIC is converted to graphene oxide, from which graphene can be obtained by reduction.^13,14^ This route tends to generate high material and processing costs. In addition, the defects that are induced in the basal plane of graphene during oxidation are not able to be fully recovered by the reduction process. Thus, in the present state, it is very challenging to develop a low-cost production route to graphene with high quality *via* graphene oxide.

With respect to electrochemical exfoliation, either cathodic or anodic potentials drive guest ions into graphene layers of the host graphite, which leads to expansion of graphite flakes. Gasification of the intercalated species then facilitates the exfoliation of the expanded graphite.^15^ Although high quality graphene can be obtained by this method, electrical connectivity to the graphite feed materials must be maintained during the entire process, and if contact is broken the graphene yield dramatically decreases. Furthermore, the high quality stock graphite materials that are required—such as rods, foil or highly oriented pyrolytic slabs—lead to high material cost. It was proposed that there is a fundamental need to re-engineer this method so that the electrochemical driving force can be applied to graphite materials more efficiently and effectively.^16^

Graphene cannot be stored in dry powder form, as mentioned above; in a liquid, however, repulsive forces such as steric and electrical repulsion are available to stabilize the graphene dispersion. A method known as liquid-phase exfoliation (LPE) was introduced in 2008, in which graphite is subjected to sonication or shear mixing in a liquid to produce few-layer graphene.^17^ Shear mixing is the simplest LPE method in terms of equipment and it is scalable,^18^ so it has strong potential for encouraging the commercialization of non-oxide graphene. To achieve good exfoliation and dispersion performance with LPE, it is imperative to choose a compatible solvent—meaning its surface tension is close to that of graphene (the surface energy is ∼68 mJ m^−2^), such that the enthalpy of mixing is minimized.^17,19,20^ Although the LPE method holds promise for the mass production of non-oxide graphene, it faces three issues that must be overcome before widespread practical application can be realized.

The most serious one is the limited selection of compatible solvents: *N*-methyl-2-pyrrolidone (NMP) and dimethylformamide (DMF) are commonly acknowledged as the most appropriate solvents, because the surface tension of these solvents matches that of most nanocarbons.^17,18^ However, drawbacks such as a high boiling point make it difficult to prepare these dispersions for subsequent processing such as ink jet printing or coating, and toxicity is also a concern for human health. For example, a typical solvent exchange process involves switching the exfoliated graphene from NMP to chloroform or alcohol;^1,2^ however, the filtration of well-dispersed graphene is time-consuming, creating a bottleneck that impedes so-called “one pot” synthesis strategies and ultimately slows down the industrial usage of graphite exfoliated *via* LPE.

The second problem is polydispersity of the thickness and a low aspect ratio. To make effective use of graphene's phenomenal properties in applications such as high-performance polymeric nanocomposites, a high aspect ratio (*i.e.*, large lateral size with a small thickness) is ideal.^21,22^ The average layer number (*N*) and aspect ratio of exfoliated graphene are usually between 3 and 7 and 50 and 100, respectively.^18^ If we separate monolayer graphene with a higher aspect ratio from few-layer graphene (FLG) with a lower aspect ratio in terms of their material properties used in engineering design, it is clear that a variable mixture of monolayer/FLG (polydispersity) would add difficulty to the design and manufacture of the final product. Thus, efficient production of graphene with uniform dimensions is ideal.

The third issue is the low yield of graphene, particularly monolayer graphene. A yield in the range of 0.1–10% after 1 h of sonication has commonly been reported, depending on the initial graphite structure and centrifugation conditions.^23,24^

Recently, microfluidization was proposed by several authors as an alternative to shear mixing or sonication.^25–27^ Microfluidization is a homogenization process in which a dispersion is forced through a narrow gap by the application of high pressure. The fluid–particle interaction inside the channel can be controlled by changing the fluid dynamics (*e.g.* turbulent flow, laminar flow, cavitation, and collision). Graphene yield using microfluidization was reportedly 10 times higher than that using high-power probe sonication.^25^ Karagiannidis *et al.* produced conductive printable graphene inks with a 100% exfoliation yield (*i.e.*, graphene rather than graphite flakes).^27^ Although it appears that the low yield of graphene by LPE might be drastically improved by microfluidization, the exfoliation degree (*i.e.*, layer number) of graphene by this method is usually insufficient; 96% of flakes fall in the 4–70 nm thickness range.^27^ Moreover, as mentioned above, the limitation of compatible solvents such as NMP and DMF systems is still the critical issue, and these facts restrict the potential of the microfluidization method and thus the application of exfoliated graphene.

**Fig. 1:**
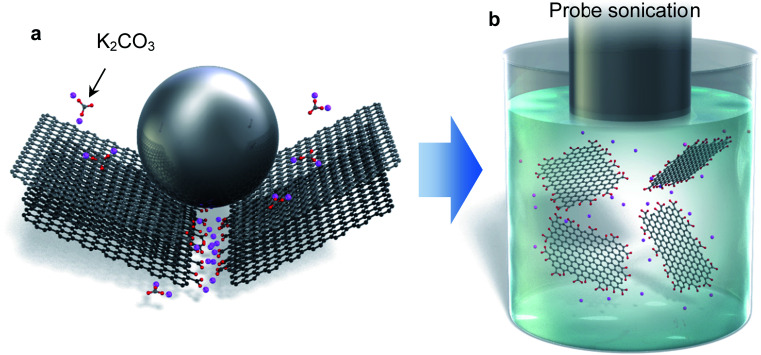
Schematic diagram of the mechanochemical reaction and exfoliation of anionic graphene. (a) Carbon radicals are generated by fragmentation of graphite by ball milling. Some salts react with the carbon radicals, forming a functionalized edge. (b) Alkali metals at the edge dissociate in a polar solvent, leading to negative charge. The enhanced electrical repulsion enables exfoliation of graphite in incompatible solvents such as water and alcohol.

Until now, there was no single method that could address all three of the above drawbacks. In this paper, we present a new modified graphite that undergoes exfoliation in low boiling point solvents to monolayer graphene, with a total yield of more than 10%. Carbon radicals induced by fragmentation of graphite in a ball milling process react with some selected salt molecules (Fig. 1a). This mechanochemical reaction produces edge-functionalized graphite that has the ability to induce negative charge in polar solvents and facilitate exfoliation in what are typically incompatible solvents, such as water, acetone, alcohol, and so on (Fig. 1b). The edge-functionalization is confirmed by detailed chemical analysis. In addition, molecular simulation reveals that the salt is adsorbed on the active carbon at the graphite edge. The soluble graphite developed in this work can definitely expand the effective usage of these advanced 2D materials.

## Results and discussion

### Mechanochemical reaction of graphite

Planetary ball milling is conducted to induce the mechanochemical reaction of graphite with salts. The approach to produce graphene *via* ball milling has been widely investigated.^28–35^ Baek's group found that edge-functionalized graphene nanoplatelets could be prepared by dry ball milling in the presence of hydrogen, carbon dioxide, or sulfur trioxide which acts as an electrocatalyst for the oxygen reduction reaction.^28,29^ Salt such as sodium chloride or sodium sulfate is added to facilitate the delamination of graphite.^30,31^ However, the crystal size of graphene gradually decreases because of the fragmentation caused by the impact of the balls, making it difficult to maintain a layered structure after a long milling time (>48 h). Because so many defects are introduced if ball milling continues for long durations, the milled graphite tends to become very fine with an amorphous structure, rather than crystalline graphene.^36,37^ To avoid this problem, a protective agent such as naphthalene, pyrene, melamine, or ammonia gas is introduced during ball milling.^32–35^ Although these additives help produce high-quality FLG, the thickness is usually not uniform and the milled powder can only be dispersed in NMP, DMF, or a water/surfactant solution.

In our process, we added weak acid salts such as potassium carbonate or sodium acetate to modify the graphite structure. The milling time, quantity of salt, and type of salt all have dominant effects on the solubility and quality of the resultant graphite; hence we carefully investigated these parameters. The functionalization process is extremely simple. The mixture of graphite and salt was milled for an arbitrary amount of time, and then the mixture was washed several times until the pH of water became neutral. The filtered cake was dried at 60 °C overnight, and then ground in a mortar to obtain soluble graphite powder. To assess the quality of the powder, Raman and XRD analyses, as well as SEM observation, were conducted. In order to investigate the solubility of graphite, 0.3 g of the produced graphite was added into 100 ml of isopropanol and sonicated for 5 min, after which the dispersion was centrifuged at 1500 rpm for 30 min to remove unexfoliated flakes. An optical absorbance *A* of the dispersion at 660 nm was measured with a spectrophotometer. The concentration of graphene C in the liquid was calculated using the Lambert–Beer law *A* = *αcl* where *l* is the light path length, *c* is the graphene concentration, and *α* is the absorption coefficient. The absorption coefficient was 3300 L g^−1^ m^−1^.The yield of graphene (*C*/*C*_i_) was calculated by dividing the output graphene concentration by the initial graphite concentration (*C*_i_ = 3 g L^−1^).

**Fig. 2:**
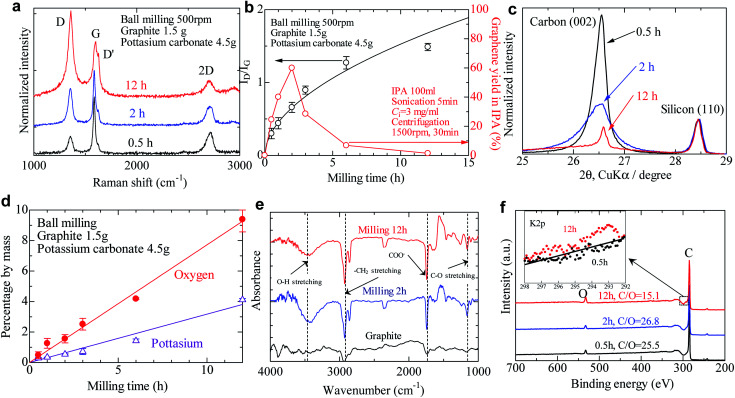
Characterization of graphite powder after salt-assisted ball milling. (a) Typical Raman spectra of graphite powders after salt-assisted ball milling. (b) Effect of milling time on the *I*_D_/*I*_G_ ratio and graphene yield after liquid-phase exfoliation in IPA. (c) XRD profiles normalized by the intensity of the silicon (110) peak. (d) Content of potassium and oxygen in graphite powder after ball milling with potassium carbonate measured by EPMA. (e) FT-IR survey of salt-assisted milled graphite. (f) XPS survey of salt-assisted milled powder. K 2p spectrum of milled powder (inset).

Typical Raman spectra of milled graphite powder are shown in Fig. 2a. It should be noted that the laser spot size was 1.0 μm, smaller than the diameter of milled graphite powder (5–30 μm, depending on the milling time), and larger than the size of individual graphene after LPE (0.4 μm on average). For characterization of graphene, we used a graphene thin film deposited on filter paper. *I*_D_/*I*_G_ values were scattered, depending on the laser position in this characterization. Therefore, we measured Raman spectra of at least 5 spots (usually 10 spots), and the average *I*_D_/*I*_G_ was obtained. This method is often used to characterize the defect or size of graphene, which has a smaller diameter than the laser spot.^18^ The spectra are normalized by the intensity of the G band (∼1590 cm^−1^). The D peak (∼1350 cm^−1^) and D′ peak (∼1620 cm^−1^), which indicate graphite defects, increased with the increasing time. The type of defects that appear in graphene—sp^3^ defects, vacancy-like defects, and edge defects—can be distinguished by the intensity ratio of the D and D′ peaks.^38^ A low *I*_D_/*I*_D′_ value of approximately 2.1 was calculated for all the milled samples, indicating that only edge defects are introduced during the ball milling process rather than basal plane defects (Fig. S1†). The intensity ratio of the D and G peaks reflects the defect density of graphite, so it is often used to quantify the quality of graphene. The *I*_D_/*I*_G_ value gradually increased with the increasing milling time (Fig. 2b), exceeding 0.5 after 2 h of milling time. The *I*_D_/*I*_G_ value of oxidized graphite is in the range of 0.8–1.5, meaning that powder milled for more than 2 h is either graphite oxide or amorphous carbon.^36,37^

Fig. 2b shows the yield of graphene by the LPE process, in which the graphene yield without salt-assisted ball milling was only 0.05% in isopropyl alcohol (IPA). This is due to the low surface tension of IPA (21.8 mN m; around 40 mN m^−1^ is preferable for exfoliation) and short sonication time. Although exfoliation was conducted in an incompatible solvent, the yield of graphene reached 25% when we used the salt-modified graphite after milling for 30 min. The yield increased to 60% when 2 h of salt-assisted ball milling was applied, but beyond 2 h the graphene yield decreased with increasing milling time—presumably due to the structural changes described above.

We varied the salt content during ball milling to study the effect of salt quantity on the resulting solubility. Milling without salt yielded 0.09%, only slightly improved compared to that of pristine graphite, while the yield increased linearly with salt content until reaching a saturated value of 30% at a salt content of 2 g (2 wt. equiv.) (Fig. S2†). It should be noted that the addition of salt did not facilitate the fragmentation of graphite. The *I*_D_/*I*_G_ value of milled powder gradually decreased with the increasing salt content (Fig. S3†) indicating that some of the milling energy was consumed by grinding the salt into finer powder, thereby reducing the energy contribution toward graphite fragmentation. The type of salt also has a dominant effect on dispersibility when using the LPE process. Inorganic (strong acid) salts such as sodium sulfate and sodium chloride are often used as a gliding assistant to improve the yield of graphene by LPE to some degree, usually between 2 and 100 times higher than that without any salt.^30^ In contrast, organic (weak acid) salts like sodium acetate, potassium sodium tartrate, and potassium carbonate improve the yield of graphene 700 times more than milling without salt (Table S1†). The reason for this difference between strong acid and weak acid salts will be discussed later in this paper. We also found that graphite powder milled with acetic acid or tartaric acid (*i.e.*, not in salt form) showed low yield (<0.03%), which points to the important role of the cation in the mechanochemical reaction and dispersion mechanisms. Salts including alkali metals (*e.g.*, potassium, sodium) improve graphene yield, whereas copper carbonate and copper phosphate have no effect on solubility of exfoliated graphene (Table S1†). Cations with a greater ionization tendency are required to improve the solubility of graphene in a solvent.

After the milled graphite strongly aggregates into spherical agglomerations after being washed and dried due to the relative strength of van der Waals forces, the size decreases with the increasing milling time (Fig. S4†). Powder XRD analysis indicates that no intercalation occurs with salt-assisted ball milling (Fig. 2c and Table S2†). The *d*-spacing of milled graphite calculated from the peak position of the (002) reflection was 0.3358–0.3364 nm, which is almost the same *d*-spacing Fig. 2b of natural graphite (0.3355). The *d*-spacing slightly increased after ball milling because the graphene structure changed from regular stacked layers to turbostratic. This value is lower than the *d*-spacing of reduced graphene oxide (0.356) or electrochemically exfoliated graphene (0.348).^39,40^ This implies that there is no intercalant and no functional group on the basal planes of graphene, which is consistent with the results of Raman analysis (defects are introduced only at the edge). The intensity of the 002 peak gradually decreased as ball milling progressed. The crystallite thickness *L*_c_ can be obtained from the full-with at half-maximum (FWHM) of the 002 profile, and the crystallite size *L*_a_ is determined from a 110 profile of carbon (Fig. S5†).^41^ The crystallite thickness decreased from 910 nm to 21.1 nm after 2 h of ball milling, meaning that the ball milling exfoliates graphite to multilayer graphene (layer number *N* > 10), but not to few-layer graphene (*N* < 10). In the case of powder treated by 12 h of milling, the *L*_c_ value could not be determined correctly because the 002 peak was asymmetric and small. The graphite structure became amorphous after 12 h of milling, with *L*_a_ = 23 nm; this might make it difficult to maintain the layered structure, leading to a spherical agglomeration (Fig. S4d†). These experimental results indicate that the powders after salt-assisted ball milling are not few-layer graphene, but simply functionalized graphite (*i.e.*, essentially no exfoliation).

To check whether potassium carbonate is chemically bonded with graphite during ball milling, electron probe micro analysis (EPMA), Fourier transform infrared spectroscopy (FT-IR), and X-ray photoelectron spectroscopy (XPS) were performed. Based on the elemental analysis of EPMA, the potassium content and oxygen content almost linearly increased with milling time. These elements gradually decreased with increasing washing time but could not be removed completely after more than five times. There are no functional groups on the graphene basal plane; thus it is reasonable to consider that the potassium carbonate adsorbs only along the edge of graphite during salt-assisted ball milling. In the FT-IR results, the characteristic peak for carboxylate (COO^−^) appears around 1720 cm^−1^ (Fig. 2e), while 1180 cm^−1^ corresponds to C–O stretching. This indicates that the carbonate ion has reacted with the ball-milled graphene to form a carboxylate-functionalized edge. Additionally, the symmetric stretching and asymmetric stretching of –CH_2_ are located at 2850 cm^−1^ and 2930 cm^−1^, respectively; the significant increase in these peaks for the ball-milled graphene is a result of passivation of the active carbons around the edges of the fractured graphene. The broad peak around 3300–3600 cm^−1^ is typical of O–H stretching, and is likely caused by the formation of alcohol or carboxylic acid groups. The C/O ratio of milled powder was approximately 25 up to 2 h of milling (Fig. 2f), but decreased to 15 after 12 h of milling. Conversely, the C/O ratio of reduced graphene is usually less than 10, indicating that the milled powder retains its original high quality for short milling times. Based on a detailed survey of the C 1s spectrum, the C=O groups increased from 2% to 9% (Fig. S6 and Table S3†) after 12 h of milling. Potassium was also detected by a detailed survey of the K 2p spectrum. These results indicate the interaction of graphite with potassium carbonate during ball milling. However, the degree of functionalization of graphite produced by salt-assisted ball milling is far less than that of traditional graphene oxide; thus it is reasonable to consider that the adsorption occurs only at the edge of the nanoplatelets.

### Liquid phase exfoliation of soluble graphite

**Fig. 3:**
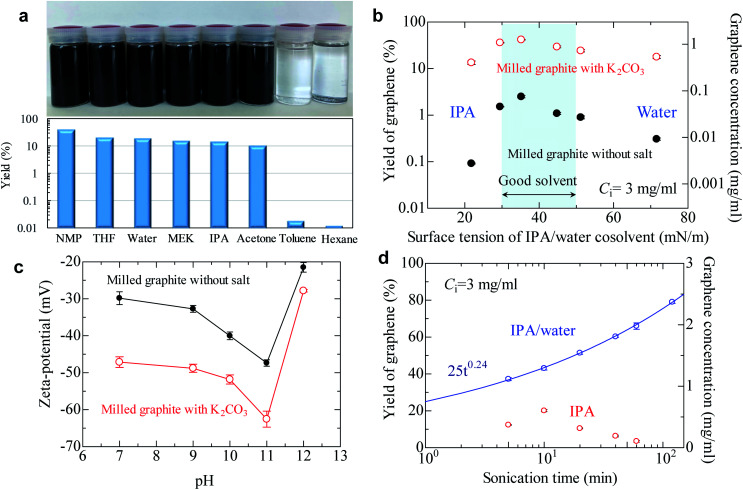
Liquid-phase exfoliation of soluble graphite. (a) Graphene yield of soluble graphite in various solvents. Yield of graphene exceeds 10% in polar solvents (dielectric constant *ε* > 5), though the concentrated dispersion cannot be obtained in nonpolar solvents (*ε* < 5). (b) Graphene yield of salt-assisted milled powder and milled powder in an IPA/water cosolvent. The surface tension of the cosolvent is controlled by changing the water content. (c) Zeta-potential of graphene as a function of pH. (d) Effect of sonication time on the graphene yield of anionic graphite. The fitting curve is obtained using the power law equation.

To produce high quality graphene, the *I*_D_/*I*_G_ should be maintained at less than 0.3—preferably less than 0.2, because the *I*_D_/*I*_G_ value reflects the defects and lateral size of graphene.^42^ Based on our comprehensive work with salt-assisted ball milling, we successfully produced soluble modified graphite with an *I*_D_/*I*_G_ of 0.21 using our method (ball milling for 20 min using 3 wt. equiv. K_2_CO_3_). To check the solubility of graphite, the modified graphite with salt was added to various solvents and then mixed with 5 min of sonication followed by 30 min of centrifugation. As shown in Fig. 3a, concentrated graphene dispersions were obtained in not only NMP but also tetrahydrofuran (THF), water, methyl ethyl ketone (MEK), IPA, and acetone. For example, the graphene concentration was 0.6 mg ml^−1^ in THF when the initial concentration of milled powder was 3 mg ml^−1^. This means the yield of graphene reaches 20% after only 5 min of sonication. The graphene concentration in acetone was 0.32 mg ml^−1^, indicating a 10% yield. Preparing concentrated graphene dispersions with these solvents is typically regarded as impossible because of the significant difference in surface tensions between the solvents and graphite. In fact, the graphene concentration was 0.0005 mg ml^−1^ in acetone when we use milled graphite without salt (Table S1†). In spite of this, the yield of graphene reached 40% in NMP and 10–20% in low-boiling point solvents. Note that the yield for milled graphite without salt in a low-boiling point solvent is less than 0.1%, which strongly reinforces the assertion that the exfoliation efficiency of graphite is significantly improved by salt-assisted ball milling. However, the modified graphite could not be exfoliated in toluene and hexane, which are non-polar solvents.

The surface tension of the solvent can be varied by controlling the water/alcohol content. The cosolvent approach is often applied to find the best surface tension of the solvent for exfoliation of layered materials. For milled graphite without salt, the best surface tension was confirmed to be around 40 mN m^−1^ (Fig. 3b), which is the same as that in previous reports.^19,20^ The soluble graphite (salt-functionalized) showed the same general trend as the milled graphite without salt, with a peak around 40 mN m^−1^ followed by a gradual decrease. However, the soluble graphite showed higher yield values compared to the non-functionalized graphite for the entire range of surface tension values. This indicates that the surface characteristics of graphene did not vary after salt-assisted ball milling, which is consistent with the Raman and XRD analysis (*i.e.*, no functional group on the basal plane).

Following from the results of Fig. 3a and b, a more dominant parameter related to a stable dispersion of soluble graphite should be discussed, in addition to the surface tension of the solvent. There are three factors to obtain a stable dispersion: compatibility of the solvent and solid (related to surface tension), steric repulsion, and electrical repulsion. The first two factors are not expected in a low boiling point solvent. Therefore, it is presumed that the electrical repulsion in a liquid is enhanced by salt-assisted ball milling. The zeta potential of graphene in a wide range of pH values was measured (Fig. 3c). The milled graphite without salt exhibited −30 mV at pH 7, which is the limiting value for a stable dispersion. While the general trend was again similar between graphite milled with and without salt, the absolute value of the zeta potential was noticeably increased by salt-assisted ball milling. The organic salt—which is chemically bonded to the graphene edges—can be dissociated in a polar solvent. When a cation such as sodium or potassium is dissociated from the edge, negative charge of the graphite is enhanced. Dissociation of salts can be determined by the conductivity of deionized water. The conductivity of deionized water increases when soluble graphite is added, whereas no conductivity change was observed for unmodified graphite (Fig. S7†). The mechanism of enhanced electrical repulsion is that cations such as sodium and potassium diffuse into the solvent, but most of them are attracted to the negatively charged particle surface. In the vicinity of the particle surface, the electrical double layer is formed. The neutralization of surface charge by the attracted cations is incomplete due to the thermal motion of said cations. Therefore, electric field around particles is negatively charged, and this generates the electrostatic repulsion between layers.

In the case of electrical repulsion, the dielectric constant *ε* of the liquid has a dominant effect on the stability of the dispersion.^43^ If the dielectric constant *ε* is sufficiently large (*ε* > 5), some electrolyte dissociation does occur which generates an electrostatic repulsion force strong enough to stabilize the dispersion. However, if *ε* < 5 then negligible dissociation occurs and the electrical double layers are extended, making it difficult to obtain an electrostatically stable dispersion system. These phenomena explain why the soluble graphite could not be exfoliated in hexane (*ε* = 1.9) or toluene (*ε* = 2.3). These solvents are categorized as non-polar solvents, so an electrical repulsion force is not expected.

The effect of sonication time on the yield of graphene was also investigated (Fig. 3d). The yield of graphene in the IPA/water cosolvent reached 80% (2.4 mg ml^−1^) after 2 h of sonication, which is the highest value obtained for the LPE process. In the case of pure IPA, a yield of 20% was obtained after 10 min of sonication. It was reported that it took 300 h to obtain a dispersion of few-layer graphene with a yield of 15% when natural graphite was used.^35^ The yield of graphene in IPA gradually decreased after more than 10 min of sonication; the reason for this phenomenon is not clear, but possibly aging of IPA might occur during sonication.^44^

**Fig. 4:**
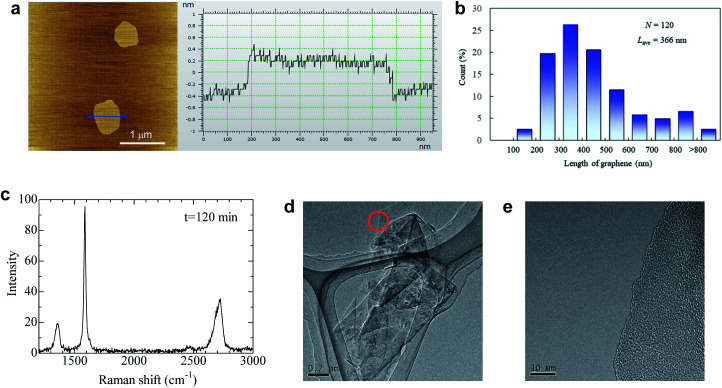
Quality of graphene after LPE using anionic graphite. (a) AFM image of graphene with its thickness profile. (b) Histogram of the graphene length in the IPA/water cosolvent measured by AFM. (c) Typical Raman spectrum of graphene deposited on the filter paper. (d) TEM image of restacked graphene sheets. (e) The magnified image of the edge marked by the circle in (d).

The quality of dispersed graphene was determined by AFM, TEM, and Raman analyses. Surprisingly, the average thickness of graphene in IPA and the IPA/water cosolvent was 0.63 and 0.59 nm, respectively, which are the smallest values obtained for liquid exfoliated graphene (Fig. 4a, S8 and S9†). The thickness of monolayer graphene measured by AFM depends on the substrate and measuring conditions, and the value is usually 0.6–1 nm.^18^ We measured the thickness of over 200 graphene nanoplatelets, and none with a thickness above 0.8 nm could be found. Thus, we assumed that nanosheets dispersed in the solvent were monolayer graphene. The proportion of monolayers was 100%. Few-layer graphene was not observed in this study, which is unusual because the centrifugation conditions were mild (1500 rpm, 30 min) and few-layer graphene is usually obtained under these conditions. We presume that the repulsive force generated by negative charge further contributes to exfoliation during sonication. The average length of graphene was 366 nm for the IPA/water cosolvent and 227 nm for the IPA solution (Fig. 4b and S10†). The IPA/water cosolvent is more compatible with graphene than IPA, so clean exfoliation frequently occurs rather than fragmentation during LPE. In an optimal solvent such as that described above, the aspect ratio of graphene was 580. In the case of LPE using natural graphite, the aspect ratio of few-layer graphene is typically 50–100.^18^ This high aspect ratio of soluble graphene will contribute to the improvement of mechanical, thermal, and barrier properties of polymer nanocomposites.^21,22^ The quality of graphene exfoliated and dispersed by the LPE process was also checked by Raman spectroscopy, and the *I*_D_/*I*_G_ value was around 0.22 by Raman analysis (Fig. 4c), which indicates high quality graphene. The value did not change even with 2 h of sonication (Fig. S11†). Thus, the quality of graphene is more influenced by the ball milling conditions than by the sonication time.

It should be noted that the agglomeration of graphene immediately occurs as the solvent evaporates because there is no repulsive force without the solvent. The isolated graphene was obtained on mica, but we could not directly observe exfoliated graphene, only its agglomeration on the Si and SiO_2_ substrate, presumably due to the weak interaction between graphene and substrates. With respect to TEM observation, graphene agglomeration was observed as shown in Fig. 4d. Only a single dark line was observed at the edge of graphene (Fig. 4e), indicating that the agglomeration is composed of randomly stacked monolayer graphene. Energy dispersive X-ray spectroscopy was performed for graphene (Fig. 4e) to check the presence of potassium at the edge of graphite; approximately 0.7 weight% potassium was detected (Fig. S12†), while no potassium was detected at the basal plane of graphite (Fig. S13†). These results indicate that the graphite edges are indeed functionalized by salt-assisted ball milling.

The productivity of graphene obtained by a combination of salt-assisted ball milling and subsequent LPE is compared with that obtained by other processes in the literature. It was reported that pyrene or melamine facilitated the exfoliation of graphite by π–π interaction with graphite.^33–35^ We have conducted the same milling process using pyrene or melamine as additives during ball milling, and the graphene yield after the LPE process was determined. The yield of graphene in acetone after centrifugation (1500 rpm, 30 min) was 0.01% for pyrene-assisted milled graphite and 1.03% for melamine-assisted milled graphite, whereas the yield of salt-assisted milled graphite exceeds 3.0% (Table S1†). These results indicate that weak acid salts are the best choice for additives in ball milling to produce soluble graphene.

### Molecular simulation

**Fig. 5:**
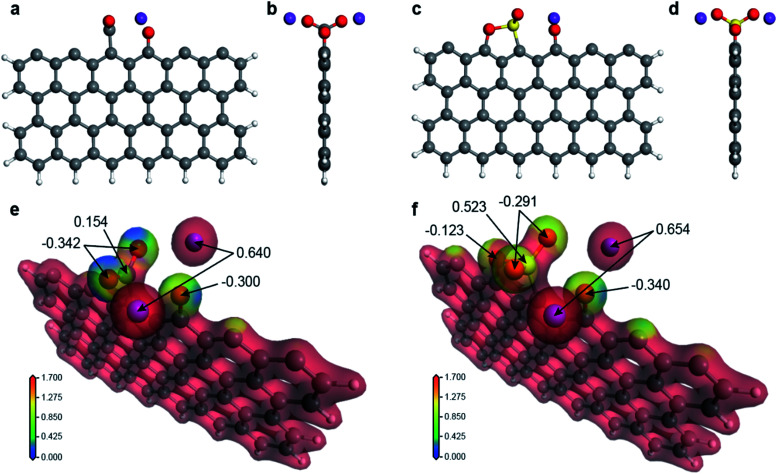
Molecular simulation of the mechanochemical reaction at the graphene edge. (a and b) Front and side views of the graphene fragment reacting with K_2_CO_3_. (c and d) Front and side views of the graphene fragment reacting with K_2_SO_4_. (e) Electrostatic potential map with the value of the Hirshfeld charge for graphene reacting with K_2_CO_3_. (f) Electrostatic potential map with the value of the Hirshfeld charge for graphene reacting with K_2_SO_4_.

It should be clarified how the active carbon at the graphite edge reacts with the salts and why weak acid salts are more efficient in improving solubility than strong acid salts. To answer the above question, we have performed a first-principles molecular simulation of a chemical reaction between a salt molecule (K_2_CO_3_, K_2_SO_4_, CH_3_COOK, and KNO_3_; K may be changed to Na) and a rectangular graphene fragment with three sides terminated by hydrogen by using DMol^3,45,46^ in Materials Studio with the DNP+ basis set and the meta-GGA functional SCAN^47^ in density functional theory. After structural optimization, we found that a –CO_2_, –SO_2_, or –NO_2_ base is adsorbed on top of one edge carbon atom in a “Y”-shape perpendicular to the graphene plane (see Fig. 5a–d for K_2_CO_3_ and K_2_SO_4_ and Fig. S14a–d† for CH_3_COOK and KNO_3_; see also the green circle in Fig. S15a–d†) with an adsorption energy more than 5 eV (see Table S4†) indicating that the final structure is energetically very stable. The electrostatic potential felt by each electron is plotted in Fig. 5e and f for K_2_CO_3_ and K_2_SO_4_ and Fig. S14e and f† for CH_3_COOK and KNO_3_ together with the value of the Hirshfeld charge, which is defined as the difference between the molecular and unrelaxed atomic charge. Obviously, the electrostatic potential of weak acid salts (blue region) is much lower than that of strong acid salts (yellow region). Additionally, the Hirshfeld charge of the Y-shaped base is much more negative for weak acid salts (typically ∼−0.5) than for strong acid salts (typically ∼−0.1). This difference is clearly due to the difference between the base atoms (C, S, or N) of the Y-shaped adsorbent; a –CO_2_ base is more negatively charged than a –SO_2_ or –NO_2_ base. This means that the original bonding nature between the alkali ion and the base molecule is retained in the case of weak acid salts, but the alkali atom ion is more strongly bonded to the graphene edge in the case of strong acid salts (see Table S4† for the Hirshfeld charge of the Y-shaped base and Table S5† for the bond length). Therefore, the alkali ions are more easily dissociated in aqueous solution and form the electrical double layer. This fact corresponds to the negatively large zeta potential of the ball milled graphene sample in aqueous solution in Fig. 3c.

## Conclusions

We have found a new mechanochemical reaction for the production of high-quality soluble graphene, whereby the activated carbon radicals induced by fragmentation react with organic salts in a pathway for edge-functionalization. In general, salt is used to precipitate a stable colloid by weakening the electrical repulsion of the colloid *via* ionic dissociation. However, if the salt is chemically bonded to the colloid, it acts instead as a dispersing agent. Dissociation of salts from the particle increases the negative charge of particles in the liquid. Thus, the salt-modified graphite can be exfoliated in low-boiling point solvents, which is commonly believed to be impossible for achieving stable dispersions. By using soluble graphite with the LPE method, essentially monolayer graphene can be obtained with a high yield (more than 10%) with just a few minutes of sonication. The process requires only common chemicals such as carbonate and acetate, which are cheap and environmentally friendly. Moreover, the process is simple (milling and washing) and scalable; thus the new mechanochemical route proposed here will be a key technology for mass production and effective usage of attractive nanomaterials.

## Methods

Natural graphite (Sigma-Aldrich, *d* = 500 mm) was used for modification by planetary ball milling (P-6, Fritsch). In most cases, seven steel balls with a diameter of 20 mm were placed in an 80 ml container. The rotation speed was controlled at 500 rpm. The milled powder was washed several times until the water pH became neutral. The washed samples were dried overnight and were ground in a mortar to obtain fine powder. Typically, 0.3 g of the powder was added into 100 ml of a solvent and probe-sonication (UH-600, SMT) was conducted to exfoliate the powder. The obtained dispersion was poured into a 50 ml centrifugation tube. The dispersion was centrifuged (model 2420, Kubota) at 1500 rpm for 30 min. The top half of the dispersion was carefully extracted using a pipette and stored for use. The characterization methods are all described in detail in the ESI.†

## Conflicts of interest

There are no conflicts to declare.

## Supplementary Material

NA-001-C9NA00463G-s001
