# Supplementary material for: Mass production of low-boiling point solvent- and water-soluble graphene by simple salt-assisted ball milling
Source: Nanoscale Adv. 2019 Nov 21;1(12):4955–64. doi: 10.1039/c9na00463g (PMC9418494; doi:10.1039/c9na00463g)
Supplement: NA-001-C9NA00463G-s001 [file NA-001-C9NA00463G-s001.pdf]

Electronic Supplementary Material (ESI) for Nanoscale Advances.
This journal is © The Royal Society of Chemistry 2019

Mass production of low-boiling point solvent- and water-soluble graphene □□□□□□□by simple salt-assisted ball milling

Authors: Yoshihiko Arao[1*], Riichi Kuwahara[2], Kaoru Ohno[3], Jonathon Tanks[1], Kojiro Aida[1], Masatoshi Kubouchi[1], Shin-ichi Takeda[4],

Affiliation:

[1] Tokyo Institute of Technology, School of Materials and Chemical Technology, 2-12-1 O-okayama, Meguro-ku, Tokyo, Japan

[2] Dassault Systèmes, ThinkPark Tower 2-1-1 Osaki, Shinagawa-ku, Tokyo, Japan

[3] Department of Physics, Yokohama National University, 79-5 Tokiwadai, Hodogaya-ku, Yokohama, Japan

4 Structures and Advanced Composite Research Unit, Japan Aerospace Exploration Agency (JAXA), 6-13-1 Osawa, Mitaka-shi, Tokyo, Japan.

1. Materials

For all experiments, we used flake graphite with large diameter (Sigma-Aldrich 332461). 2-propanol (IPA), acetone,tetrahydrofuran (THF), ethyl methyl ketone (MEK), toluene and hexane were purchased from Kanto Chemical. Salts including sodium sulfate, sodium nitrate, magnesium sulfate, sodium chloride, monosodium glutamate, sodium acetate, potassium acetate, diammonium tartrate, potassium sodium tartrate, tripotassium phosphate, copper phosphate, potassium carbonate, sodium carbonate and copper carbonate were purchased from Kanto Chemical and Wako Chemical. Acetic acid and tartaric acid were purchased from Kanto Chemical.

2. Ball milling condition

In our experiment, planetary ball milling (P-6, Fritsch) was used to facilitate the mechanical reaction of graphite with salts. Ball milling condition should be carefully selected. We found that impact mode of ball milling is more suited than friction mode for mechanochemical reaction. There are two important factors to fragment graphite: ball size and number of ball. Smaller ball size is preferable, because frequency of impact between balls dramatically increase with decreasing the size of ball. However, the crashing energy per one impact also decrease, and fragmentation of graphite unlikely occurs when the size of ball is smaller than the threshold value. The threshold value we confirmed was in the range of 1 to 5 mm of diameter, depending on the rotation speed. To ensure mechanochemical reactions occur, we choose steel balls with a diameter of 20 mm in most cases. The mode of ball (impact or friction) depends on the fill factor of balls in a container. If five balls are added, balls are rotated inside the container rather than impact. We cannot check the ball movement visually, but estimate the mode by sound during milling. In the case of impact mode, big sound of impact is heard, in contrast small sliding sound is heard for shear sliding. The mode is changed from shear sliding to impact, when we added the balls more than 6. In the case of shear sliding, the graphite might be exfoliated, but solubility of graphite (yield of graphite in LPE process) is worse than the graphite milled at impact mode. Thus, seven balls with 20 mm diameter was usually added into a container to occur impact of balls. These condition (size of ball and filling factor of ball) depends on the machine and volume of container, and should be determined by experimental try and error in the case of planetary ball milling. With respect to the tumbling milling, there is an equation to calculate the critical rotational speed that can avoid the corotation of balls at walls of container:

$$N_C = \frac{60}{\pi}\sqrt{\frac{g}{2(d_M - d_B)}} = \frac{42.3}{\sqrt{d_M - d}_B}, \quad (1)$$

where d_m is an inner diameter of container and d_B is a diameter of ball.

3. Characterization

The size of synthesized powder was precisely measured using SEM (JSM6510-LA, JEOL Ltd.). XRD (X'pert-MPD-OES, PANalytical) was used to measure the crystallite size (L_a), the crystallite thickness (L_c), and the *d*-space of graphite, using 10 mass% of silicon powder (SRM 640e, NIST) as reference powder for XRD measurement. CuKa radiation was used for measurement. Excitation voltage and electric current were maintained at 45 kV and 40 mA, respectively. Element analysis was performed by EPMA (JXA-8200, JEOL Ltd.). XPS (K-ALPHA, Thermo Scientific) and FT-IR (FTIR-8300, Shimadzu) measurements were also performed to characterize the synthesized powders. For XPS measurement, the surface of the powder was removed by argon etching for 110 s. Quality of synthesized powder and graphene after LPE process was characterized by Raman spectroscopy (NRS-4100, JASCO Co.) using 532 nm laser. The measurement was conducted more than five times and average values of I_D/I_G were determined. The graphene concentration of the dispersion was measured by optical absorbance at 660 nm using spectrophotometer (ASV11D, Shimadzu). At least three dispersions were checked to obtain average yield of graphene in LPE process. The size of nanosheet was measured using AFM (SPM-9700, Shimadzu) set to dynamic tapping mode. Zeta potential measurements of FLG in water were taken by nano Partica SZ-100 (Horiba, Ltd.). First, 1 ml of centrifuged dispersion was diluted by pH controlled water more than 10 times, then the diluted solution was poured into the cell for zeta potential measurement. The measurement was repeated three times and an average value of zeta potential was determined. Detail method was described below.

3.1 XRD analysis

For XRD measurement, we followed to a standard procedure of X-ray diffraction mesuremant on carbon materials[1]. The peak profiles obtained by conventional XRD measurement depend on sample preparation and measurement condition. The key point of the standard process is adding 10 mass% of

silicon powder (SRM 640e, NIST). By comparing with both the carbon profile and silicon profile, the d-spacing, L_a, and L_c values of the graphite sample were determined. CuKa radiation was used for measurement. Excitation voltage and electric current were maintained at 45 kV and 40 mA, respectively.

The d_{002} was determined using Bragg's equation below:

$$d_{002} = \lambda / \sin\theta_C \,, \qquad (2)$$

where λ is the wave length of CuKa radiation (=0.152186661 nm), and θ_c is the diffraction angle of graphite at C(002). L_a obtained from the full width half maxim (FWHM), *B,* of C(110) (graphite) and FWHM, *b,* of Si(331) (silicon). The true FWHM, β, was determined using below equation

$$\beta / B = 0.9981266 - 0.0681532 \cdot \nu - 2.592769 \cdot \nu^2 + 2.621163 \cdot \nu^3 - 0.9584715 \cdot \nu^4 \,, \qquad (3)$$

where $\nu = b / B$. In the case of L_c, FWHM *B* at C(002) and FWHM *b* at Si(110) were used. L_a and L_c were calculated by substituting the β value into the equation below:

$$L = K\lambda / \beta \cos\theta_C \qquad (3)$$

The shape factor, *K,* in Scherrer's equation is assumed to be 1.

3.2 AFM measurement

It is challenging to deposit nanosheets in a dispersion onto substrates without aggregation occurring. In our process, the dispersions (IPA or IPA/water cosolvent) were diluted with IPA to realize the momentary evaporating of solvent during spray coating. The optical absorbance of the diluent was controlled approximately A = 0.2. Bath sonication was applied for 1 min, then the dispersion was spray-coated onto freshly cleaved mica heated at 180 ˚C by a hot plate. By this method, the graphene without restacking was observed by AFM. It should be noted that we could not deposit graphene on Si and Si/SiO_2 wafers (including 100nm of oxide layer) without agglomeration by above method. This is presumably due to the weak interaction between graphene surface and Si wafers.

3.3 Conductivity of solution

In order check dissociation of soluble graphite in water, conductivity of deionized water with soluble graphite was measured. Proper amount of graphite was added into 100 ml of deionized water, then probe-sonication was conducted for 5 min. The electrical resistivity R of the dispersion was measured by bridge circuit method. By this method, high electrical resistivity of deionized water can be precisely measured. The probe with a cell constant C_0 of 95 m^{-1} was used for the measurement. The temperature of dispersion was stabilized at 25 ˚C, then electrical resistivity of the dispersion was measured at least twice. The conductivity of dispersion was determined by multiplying cell constant and electrical resistivity.

4. Molecular simulation

The results of a first-principles molecular simulation of a chemical reaction between a salt molecule (CH_3COOK and KNO_3) and a rectangular-shape graphene fragment with three sides terminated by hydrogen are shown here. The data for Na_2CO_3 and Na_2SO_4 are also shown here. The simulation method is explained in the main article. After structural optimization, we found that a -CO_2 , -SO_2, or -NO_2 base is absorbed on top of one edge carbon atom in "Y"-shape perpendicular to the graphene plane (see Fig. 5a-d for K_2CO_3 and K_2SO_4 and Fig. S14a-d for CH_3COOK and KNO_3) with the adsorption energy more than 5 eV; see Table S4. The electrostatic potential felt by each electron is plotted in Fig. 5e,f for K_2CO_3 and K_2SO_4 and Fig. S14e,f for CH_3COOK and KNO_3 together with the value of the Hirshfeld charge. Obviously, the electrostatic potential of weak acid salts (blue region) is much lower than that of strong acid salts (yellow region). As well, the Hirshfeld charge of the Y-shape base is negatively much larger for weak acid salts (typically ~ -0.5) than for strong acid salts (typically ~ -0.1; see Table S4. The bond length between the alkali atom ion and the base molecule is shorter in the case of weak acid salts and the alkali atom ion is more strongly bonded to the graphene edge in the case of strong acid salts; see Table S5.

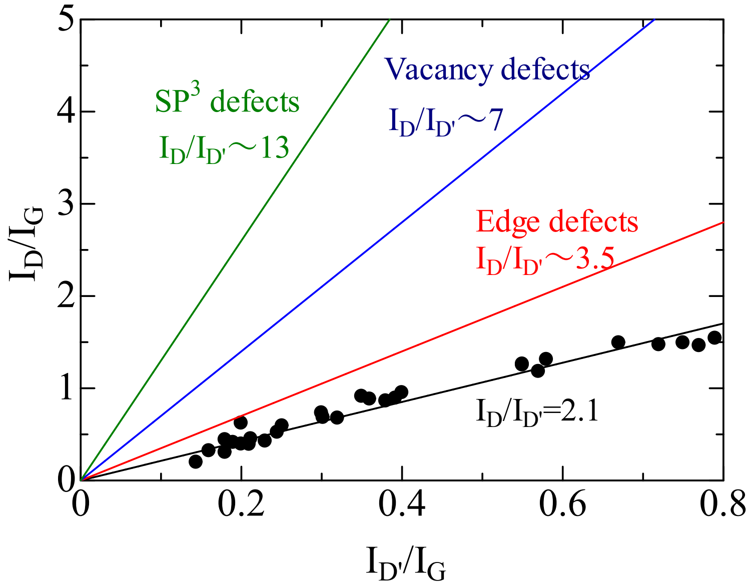

Figure S1: I_D/I_G versus ratio $I_{D'}/I_G$. Low value of I_D/I_D’ (2.1) indicates the milled graphite has boundary defect without any vacancy or SP^3 defects.

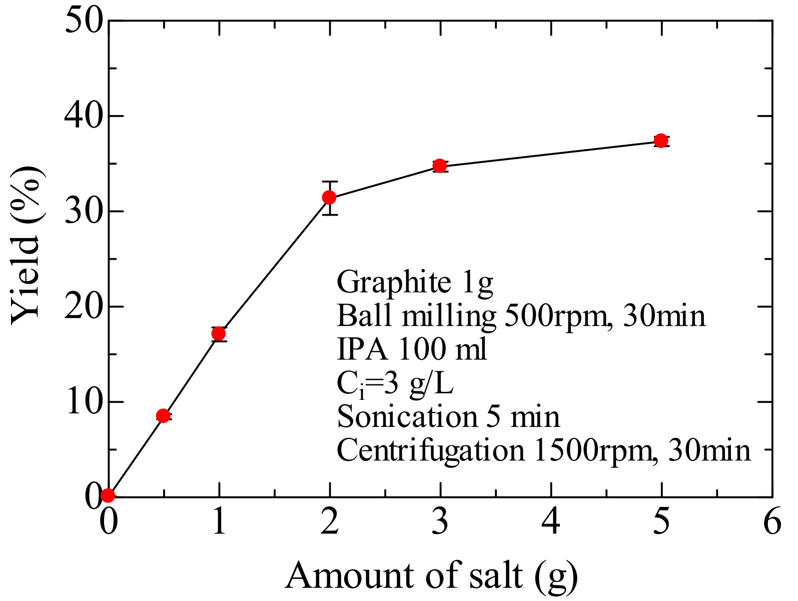

Fig. S2: Effect of salt content (K_2CO_3) on the graphene yield after liquid-phase exfoliation and centrifugation.

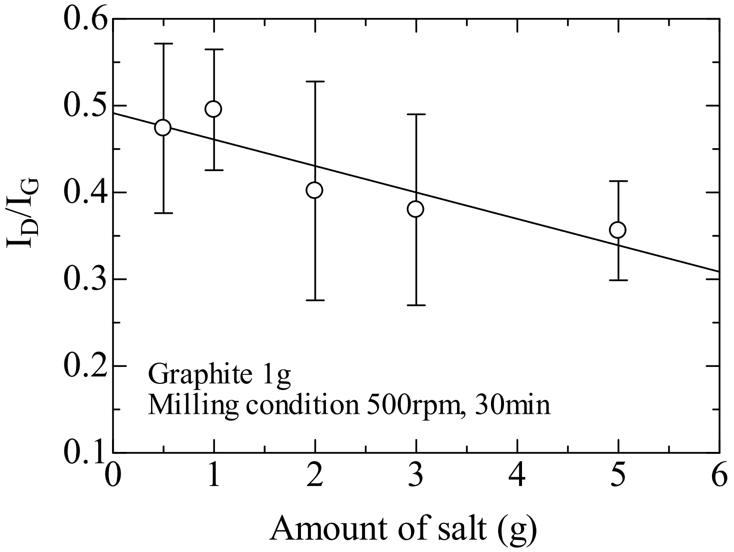

Figure S3: I_D/I_G ratio of graphite powders after 30 min of ball milling with various salt content (K_2CO_3).

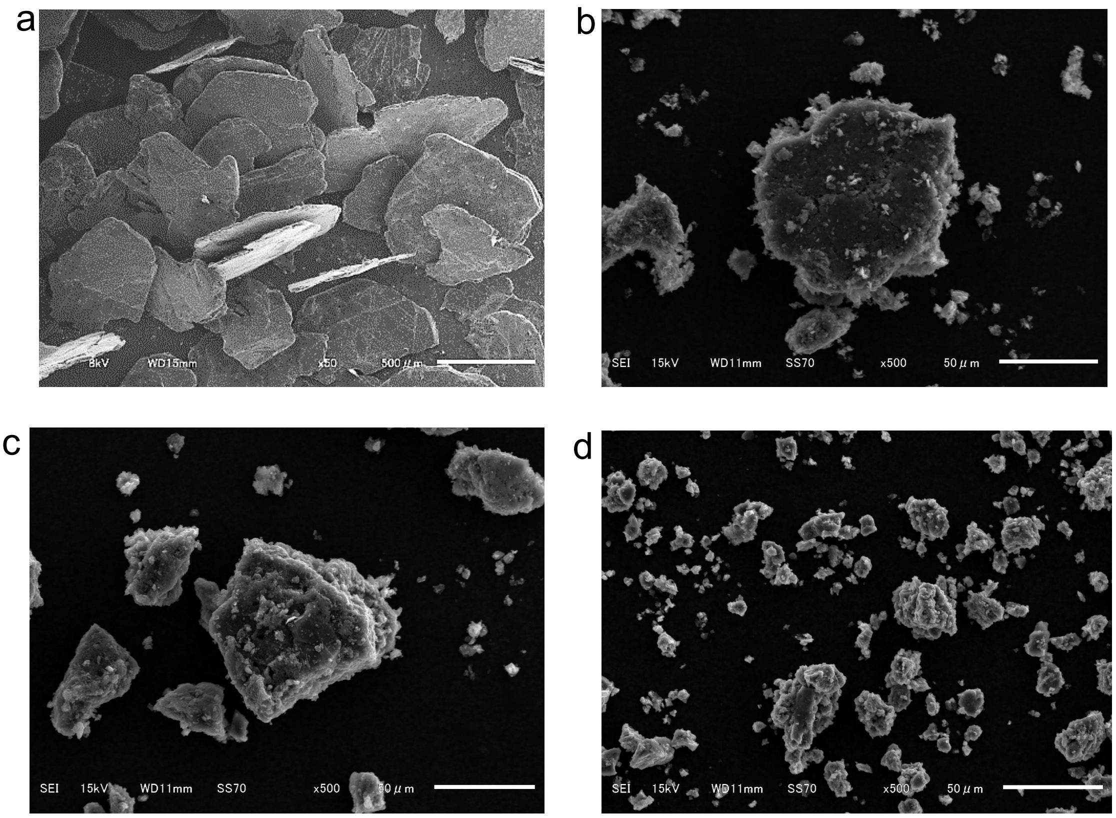

Figure S4: Graphite powders observed by SEM. a, as-received graphite. **b**, 0.5 h milling. **c**, 2 h milling. **d**, 12 h milling.

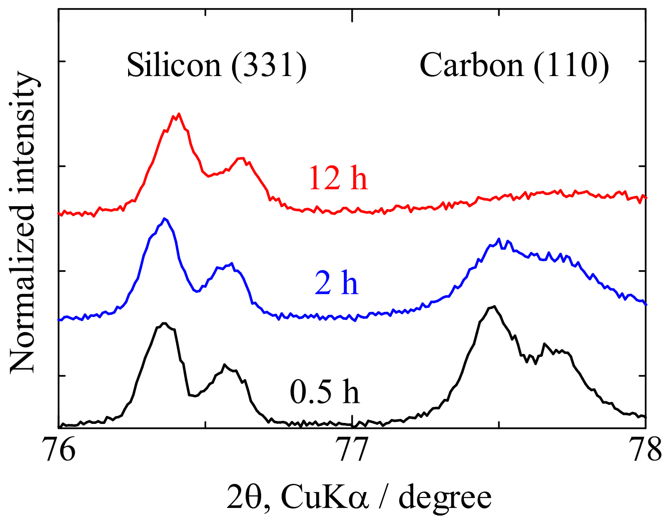

Figure S5: XRD profiles of carbon (110). All profiles are normalized with an intensity of silicon (331) peak.

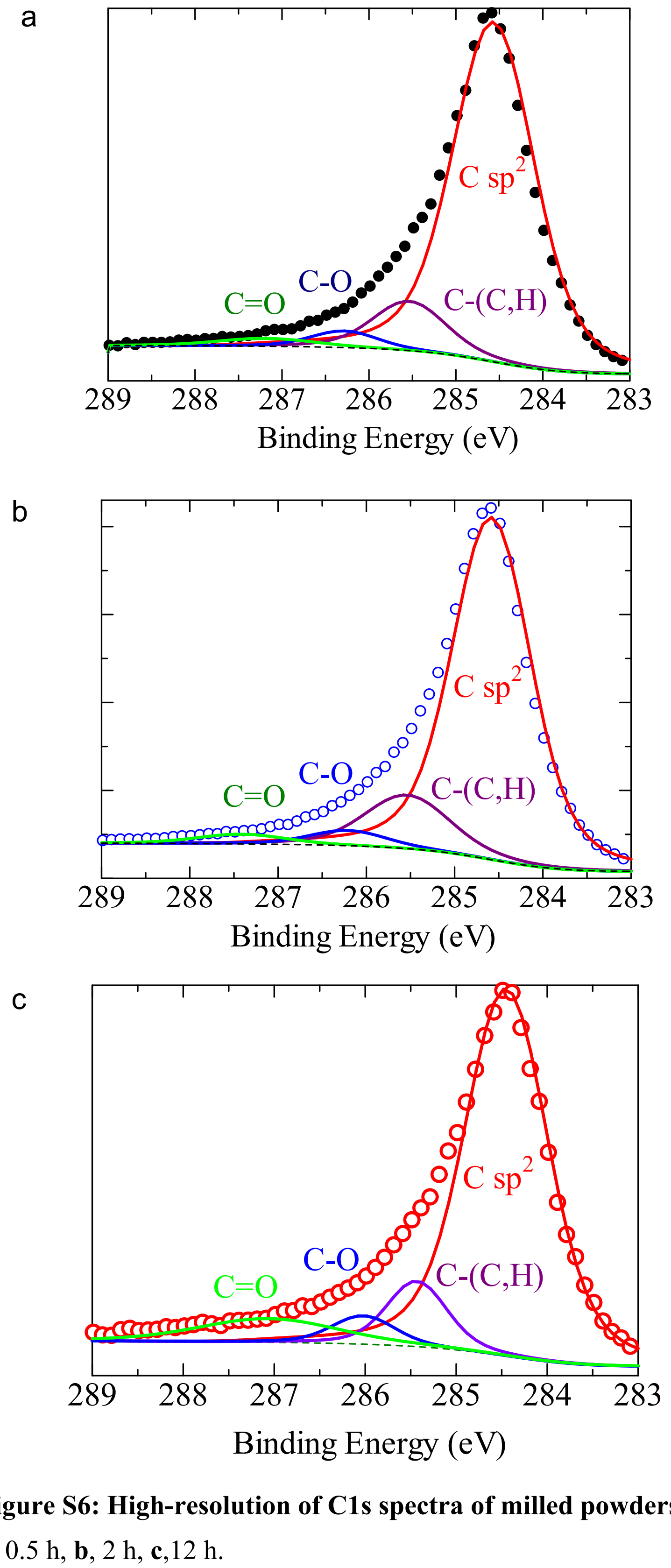

Figure S6: High-resolution of C1s spectra of milled powders.

a, 0.5 h, **b**, 2 h, **c**,12 h.

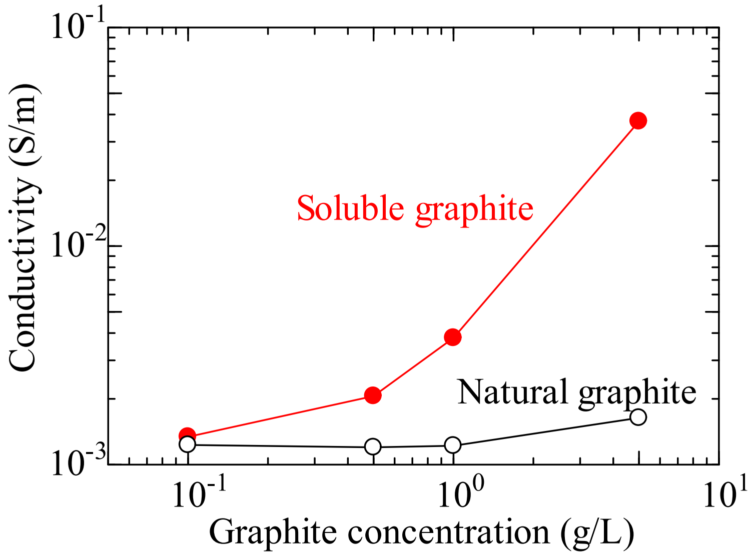

Figure S7: Conductivity changes of deionized water as a function of graphite concentration. Bridge circuit system was constructed to measure electrical resistivity.

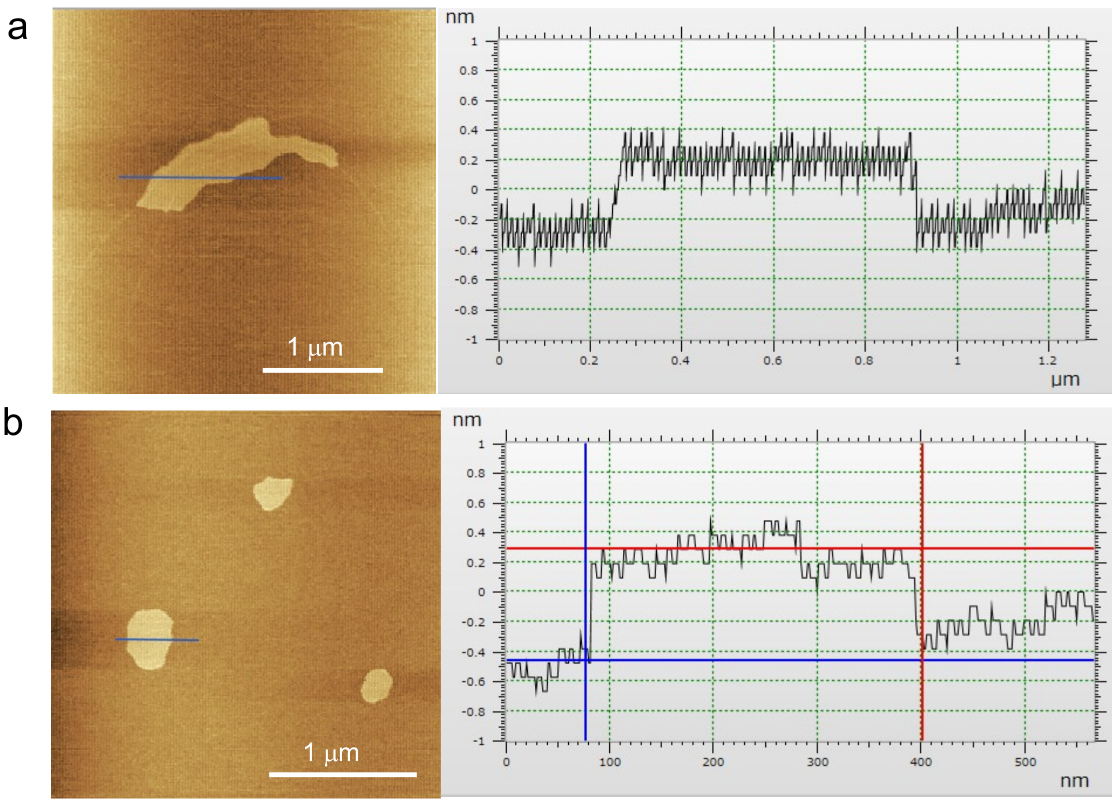

Figure S8: Additional AFM images. a, A graphene with a length more than 1μm. **b**, Typical graphene images.

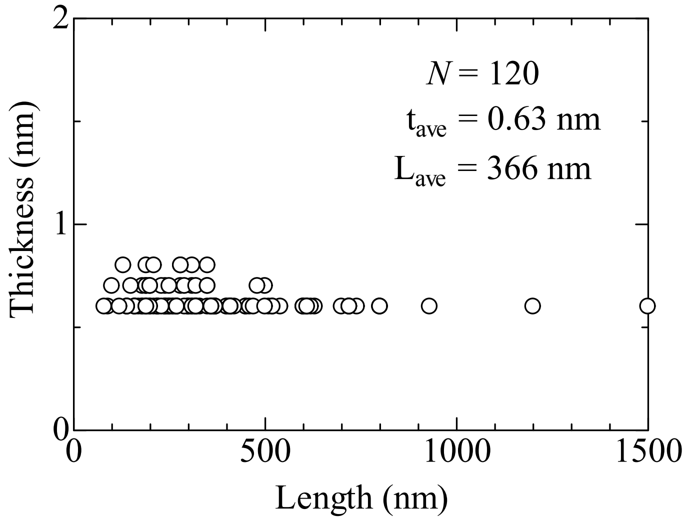

Figure S9: Plot of nanosheet thickness vs. length for soluble graphene in IPA/water cosolvent (40/60 vol.%).

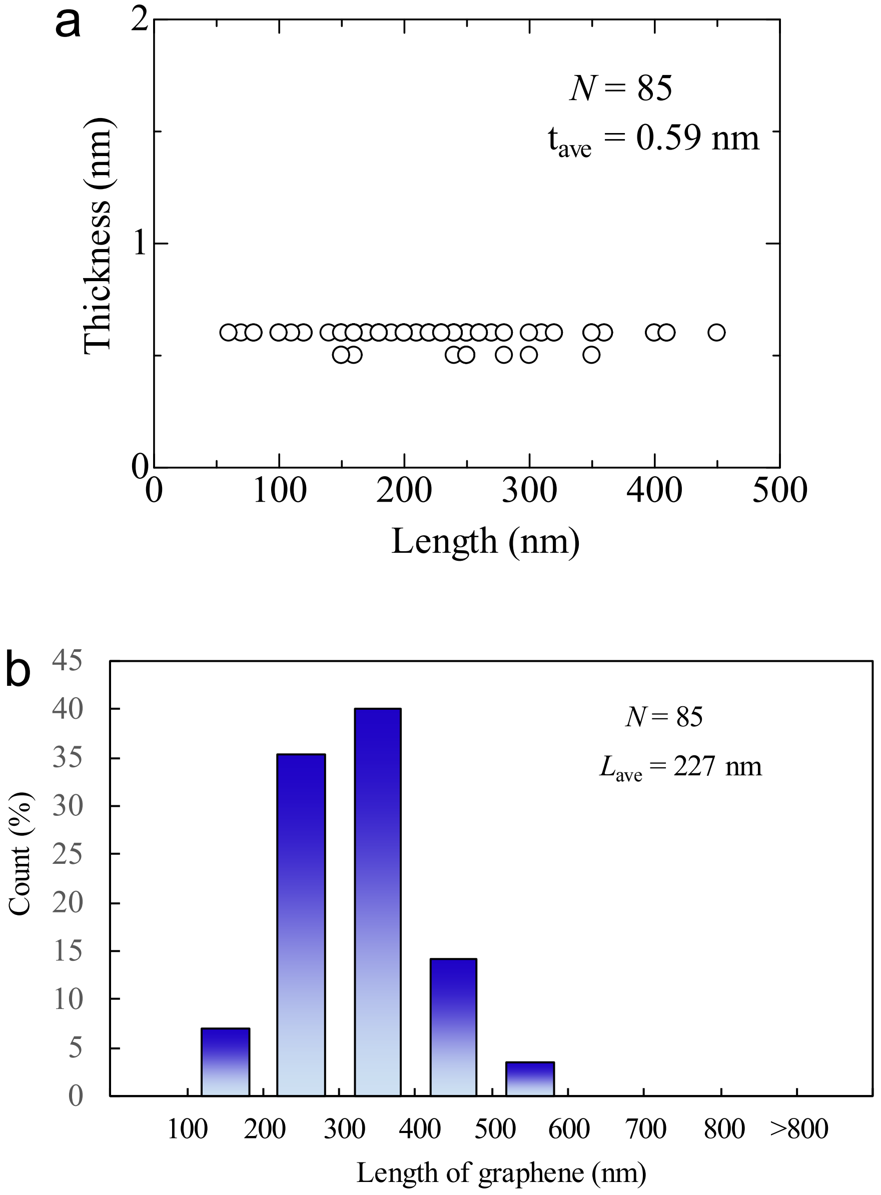

Figure S10: Quality of graphene in IPA. a, Plot of nanosheet thickness vs. length for soluble graphene in IPA. **b**, Histogram of graphene length.

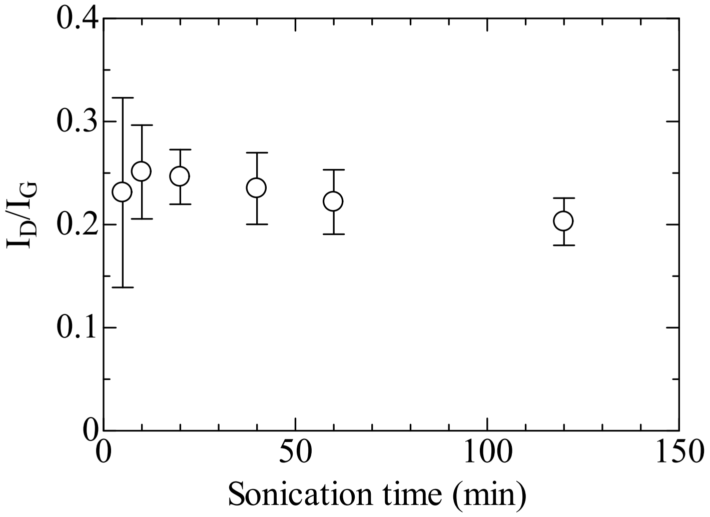

Figure S11: I_D/I_G ratio of soluble graphene as a function of sonication time.

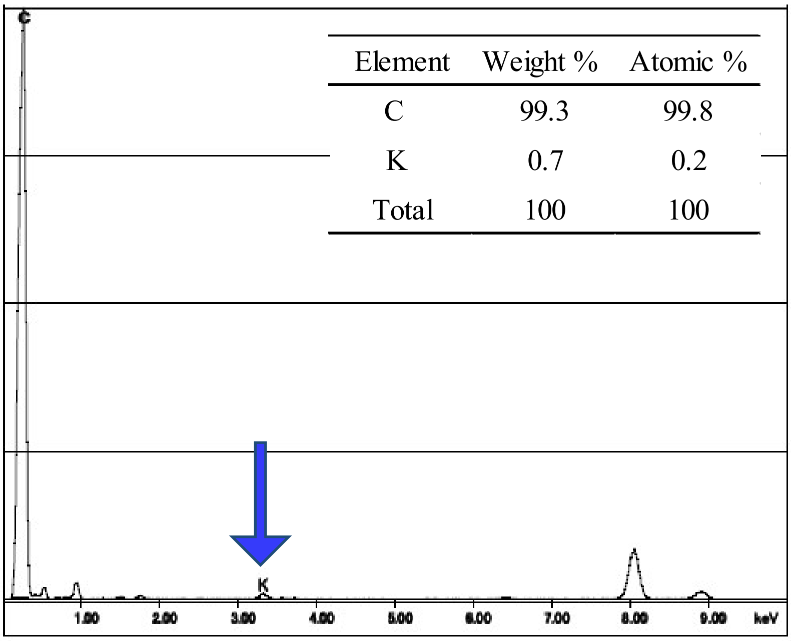

Figure S12: EDS spectrum of graphene edge (Fig. 5e).

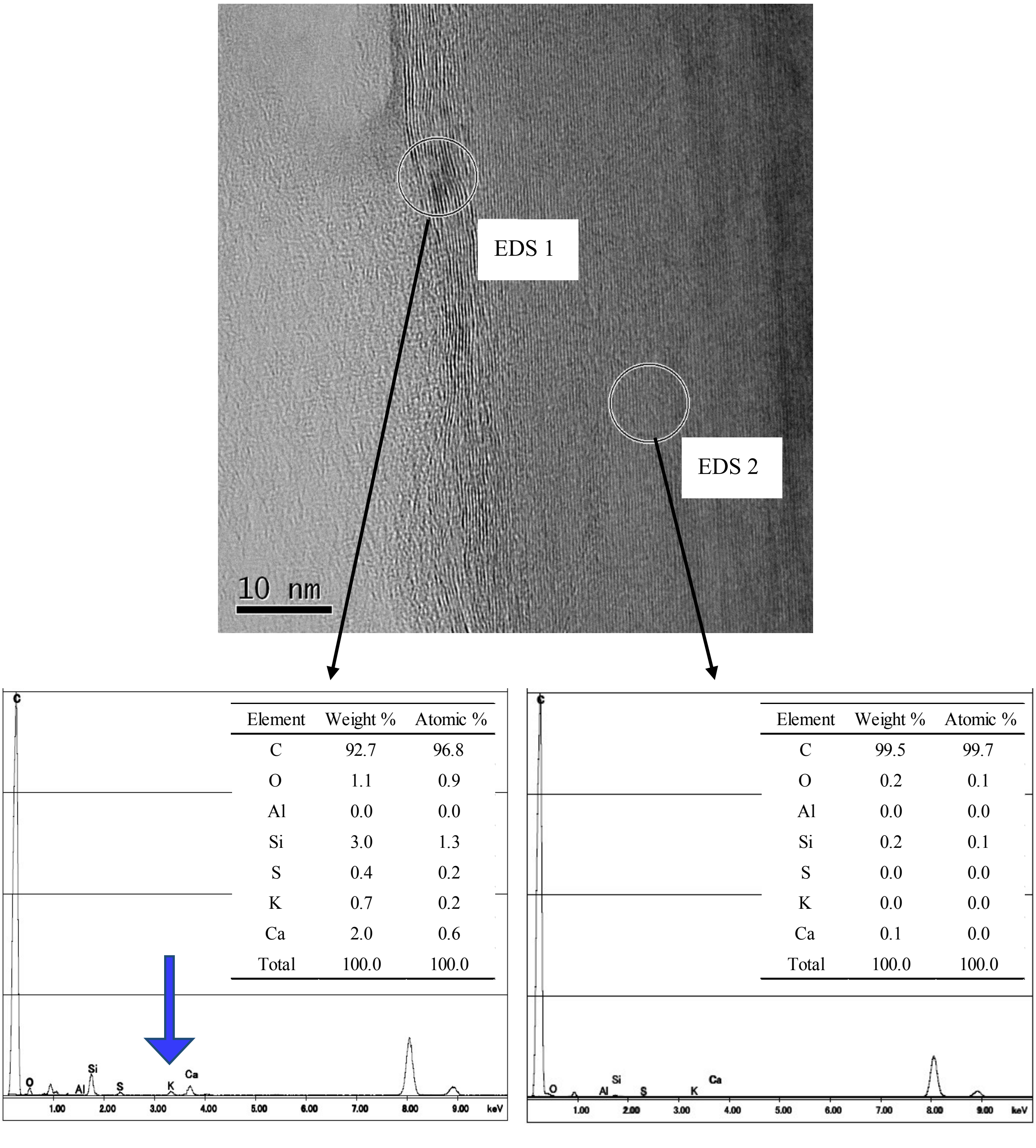

EDS 1:

Element	Weight %	Atomic %
C	92.7	96.8
O	1.1	0.9
Al	0.0	0.0
Si	3.0	1.3
S	0.4	0.2
K	0.7	0.2
Ca	2.0	0.6
Total	100.0	100.0

EDS 2:

Element	Weight %	Atomic %
C	99.5	99.7
O	0.2	0.1
Al	0.0	0.0
Si	0.2	0.1
S	0.0	0.0
K	0.0	0.0
Ca	0.1	0.0
Total	100.0	100.0

Figure S13: HRTEM images of soluble graphite and EDS spectrum at edge (EDS 1) and basal plane (EDS 2). Potassium was detected only at the edge of soluble graphite. At the black spot (EDS 1) some impurities such as Si and Ca was also detected.

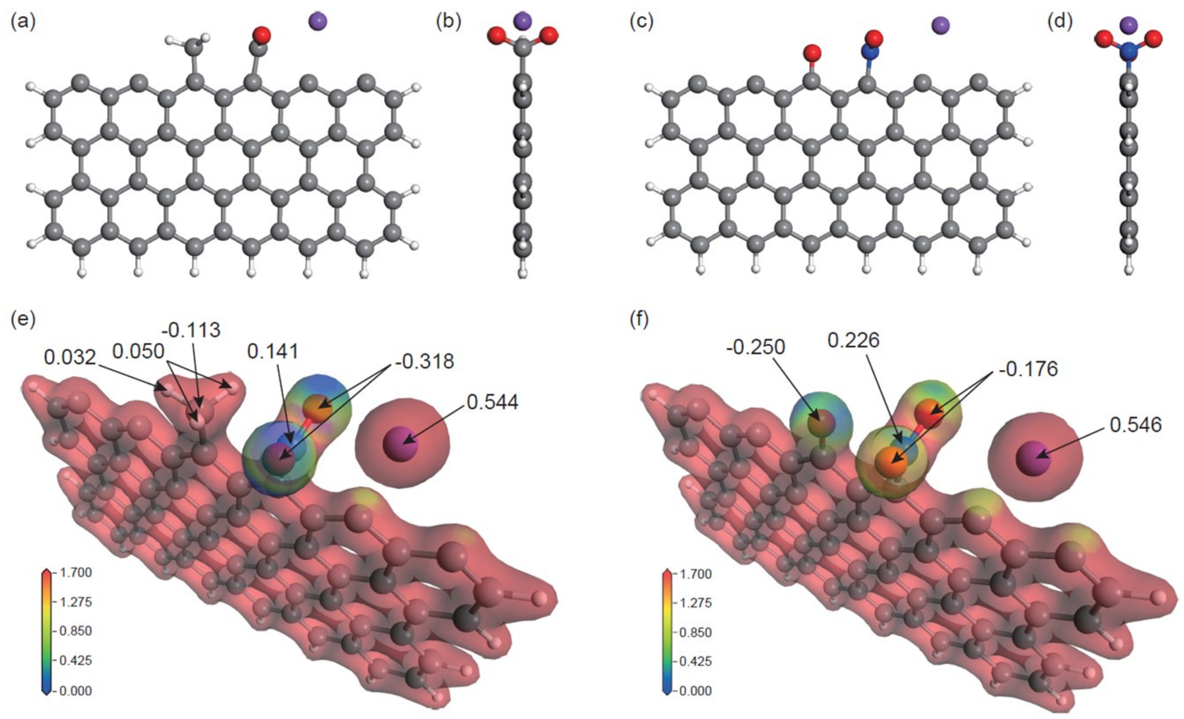

Figure S14: Molecular simulation of mechanochemical reaction at graphene edge. **a,b**, Front and side views of graphene fragment reacted with CH_3COOK. **c, d**, Front and side views of graphene fragment reacted with KNO_3. **e**, Electrostatic potential mapping together with the value of the Hirshfeld charge for graphene reacted with CH_3COOK. **f**, Electrostatic potential mapping together with the value of the Hirshfeld charge for graphene reacted with KNO_3.

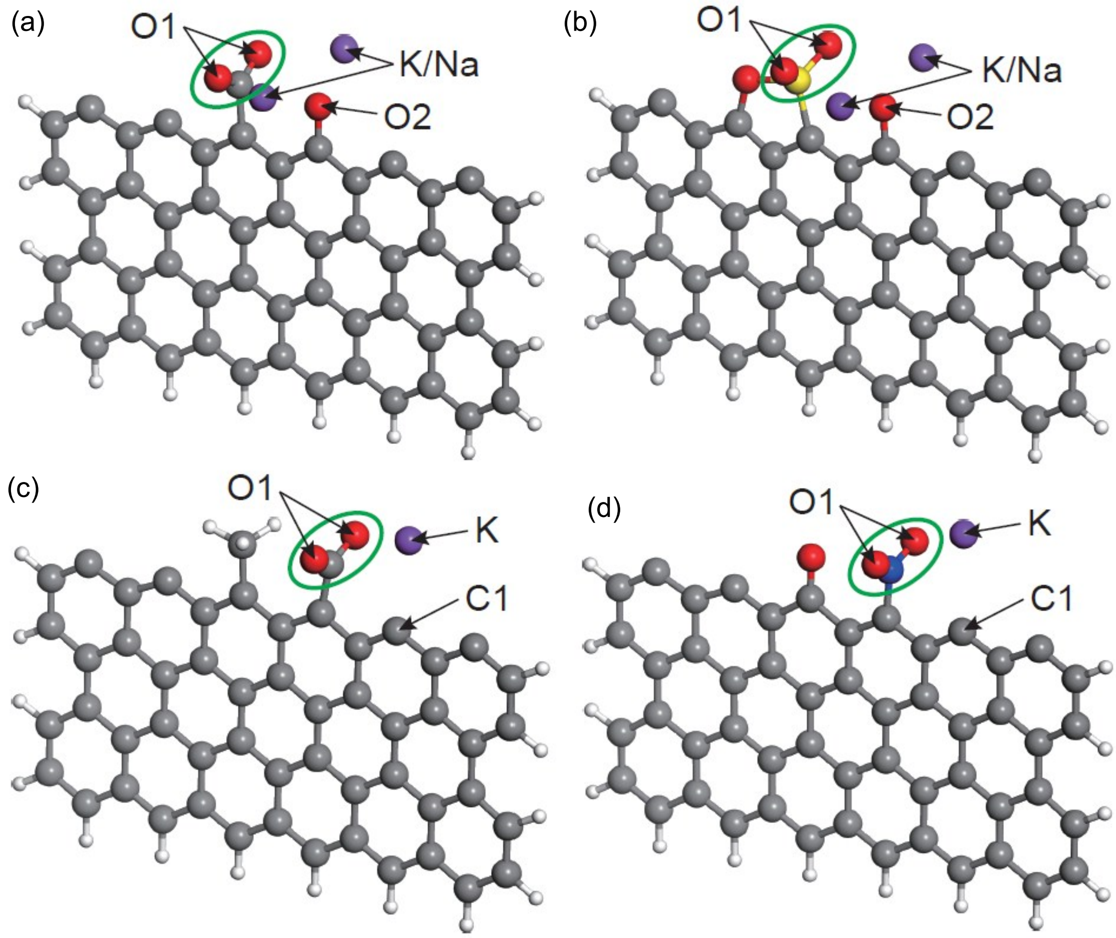

Figure S15: Atom index of graphene fragments. Green circles indicate the Y-shape adsorbed molecules related with ion bonding with alkali metals. **a**, K_2CO_3, **b**, K_2SO_4, **c**, CH_3COOK, **d**, KNO_3.

Table S1: Concentration of graphene in acetone dispersion after 5 min of sonication using various type of ball-milled graphite. In this screening, 2 g of natural graphite and the same amount salt was milled for 30 min at 500 rpm. 1 g of treated graphite was added into 100 ml of acetone, then sonicated for 5 min. The unexfoliated graphite was separated by centrifugation at 1500 rpm for 30 min. The optical absorbance of supernatant was measured to determine the graphene concentration of dispersion.

Solvent	Additive during ball milling	Composition	Classification	Concentration (g/*l*)	Yield▯ (%)	Solubility
	None	-	-	0.0005	0.005	×
	Sodium sulfate	Na_2SO_4		0.0009	0.009	×
	Sodium nitrate	$NaNO_3$	Strong acid salt	0.0005	0.005	×
	Magnesium sulfate	$MgSO_4$		0.0012	0.012	×
	Sodium chloride	NaCl		0.0738	0.738	▯
	Monosodiumu glutamate	$C_5H_8NO_4Na$		0.2059	2.059	▯
Acetone	Sodium acetate	CH_3COONa		0.3284	3.284	▯
	Pottasium acetate	CH_3COOK		0.2641	2.641	▯
	Diammonium tartrate	$C_4H_{12}N_2O_6$		0.1147	1.147	▯
	Potassium sodium tartrate	$C_4H_4KNaO_6$▯ $4H_2O$	Weak acid salt	0.2706	2.706	▯
	Tripotassium phosphate	K_3PO_4		0.2435	2.435	▯
	Copper phosphate	$Cu_3(PO_4)_2 \cdot H_2O$		0.0009	0.009	×
	Potassium carbonate	K_2CO_3		0.3294	3.294	▯
	Sodium carbonate	Na_2CO_3		0.2953	2.953	▯
	Copper carbonate	$CuCO_3$		0.0040	0.040	×
	Tartaric acid	$C_4H_6O_6$	Weak acid	0.0014	0.014	×
	Acetic adid	CH_3COOH		0.0013	0.013	×
	Pyrene (washed with acetone)		non-covalent interaction	0.001	0.01	×
	Melamine (washed with hot water)			0.1029	1.029	△

Table S2: Structures of graphite after salt-assist ball milling. *d*-spacing, crystallite thickness L_c and crystallite size L_a are determined based a standard procedure of X-ray diffraction measurements on carbon materials.

Milling time (h)	002 reflection				110 reflection
	$2\theta_c$ (°)	d_{002} (nm)	FWHM(°)	L_c(nm)	L_a(nm)
0	26.561	0.3355	0.142	910	> 1000 (1578)
0.5	26.523	0.3358	0.293	69.6	317
2	26.469	0.3364	0.577	21.1	177
12	26.561	0.3355	-	-	23

Table S3: The amount of each element for salt-assist milled graphite obtained from XPS spectra.

	Atomic %					
	0.5 h		2 h		12 h	
K2p	0.08		0.2		0.44	
O1s	3.7		3.52		6.05	
Si2p	0.7		0.3		0.33	
Fe2p	0		0		0	
Al2p	1.15		1.68		1.86	
C1s (C_sp^2)	78.77	94.4	75.76	94.3	69.34	91.3
C1s (C-C,H)	10.27		12.8		8.89	
C1s (C-O)	3.15		3.3		4.07	
C1s (C=O)	2.17		2.44		9.03	
C/O	25.5		26.8		15.1	

Table S4: Total adsorption energy of the salt (K_2CO_3, K_2SO_4, Na_2CO_3, Na_2SO_4, CH_3COOK, and KNO_3;) on the open edge of the rectangular-shape graphene fragment in units of eV and the Hirshfeld charge of the adsorbed Y-shape (-CO_2, -SO_2, and -NO_2) base.

CO_3	K_2	Na_2
Total adsorption energy of the salt (eV)	5.577	5.380
Hirshfeld charge of the -CO_2 base	-0.530	-0.507

SO_4	K_2	Na_2
Total adsorption energy of the salt (eV)	7.141	6.877
Hirshfeld charge of the -SO_2 base	-0.068	-0.062

CH_3COO	K
Total adsorption energy of the salt (eV)	5.605
Hirshfeld charge of the -CO_2 base	-0.495

NO_3	K
Total adsorption energy of the salt (eV)	6.416
Hirshfeld charge of the -NO_2 base	-0.124

Table S5: The bond length of each atomic. The atomic indices are shown in Fig. S15. In the case of weak acid (CO_3 and CH_3COO), potassium (or sodium) is close to O1, which is the –CO_2 base, indicating that the original bonding nature between the alkali atom ion and the base molecule is kept. On the other hand, the potassium of strong acid salt (SO_4 and NO_3) is close to O2 or C1 atom. It means the alkali atom ion is more strongly bonded to the graphene edge.

Bond	Length (Å)			
	CO_3	SO_4	CH_3COO	NO_3
K - O1	2.445	2.700	2.706	3.183
K - O2	2.524	2.439	N/A	N/A
K - C1	N/A	N/A	2.758	2.649

Bond	Length (Å)	
	CO_3	SO_4
Na - O1	2.113	2.262
Na - O2	2.199	2.143

Reference

[1] Iwashita N, Park CR, Fujimoto H, Shiraishi M, Inagaki M. Specification for a standard procedure of X-ray diffraction measurements on carbon materials. Carbon, Vol. 42, 701-714, 2004.
